# Systematic review and meta-analysis of the prognostic significance of microRNAs in cervical cancer

**DOI:** 10.18632/oncotarget.23839

**Published:** 2017-12-16

**Authors:** Zhilan Chen, Yingyan Han, Chengwen Song, Huafang Wei, Ying Chen, Kecheng Huang, Shuang Li, Ding Ma, Shixuan Wang, Jing Wang, Qiping Lu

**Affiliations:** ^1^ Department of Obstetrics and Gynecology, Wuhan General Hospital of People's Liberation Army of China, Wuhan, Hubei, China; ^2^ Department of Obstetrics and Gynecology, Tongji Hospital, Tongji Medical College, Huazhong University of Science and Technology, Wuhan, Hubei, China; ^3^ Department of General Surgery, Wuhan General Hospital of People's Liberation Army of China, Wuhan, Hubei, China

**Keywords:** cervical cancer, miRNA, prognosis, meta-analysis

## Abstract

In this meta-analysis, we analyzed case-control studies that assessed the prognostic potential of miRNAs in cervical cancer. We comprehensively searched EMBASE and PubMed databases and enrolled seven studies with 445 cervical cancer cases. A fixed effects model was used to calculate pooled hazard ratios (HRs) and associated 95% confidence intervals (95% CIs) from the overall survival (OS) data. Our analysis showed that poor OS in cervical cancer was associated with low miR-125 expression (HR = 1.61, 95% CI: 1.02–2.55, *P* = 0.042; I^2^ = 10.1%, *P* = 0.292; *n* = 99), low miR-145 expression (HR = 1.70, 95% CI: 1.29–2.24, *P* < 0.001; I^2^ = 0%, *P* = 0.560; *n* = 193) and high miR-196 expression (HR = 0.28, 95% CI: 0.15–0.52, *P* < 0.001; I^2^ = 0%, *P* = 0.950, *n* = 197). This makes microRNAs such as miR-125, miR-145 and miR-196 potential prognostic biomarkers in cervical cancer.

## INTRODUCTION

Cervical cancer is the third most common malignancy in females and accounts for 12% of all cancers globally [1]. More than 200,000 women die from cervical cancer each year [2]. The incidence of cervical cancer is about six times higher in China than in other developed countries [3]. Therefore, identifying new and efficient prognostic biomarkers are important objectives of cervical cancer research. There is increasing evidence that miRNA expression is abberant in human cancers [4, 5]. Moreover, miRNA expression signatures are associated with clinical outcomes of many diseases [6, 7]. MiRNAs are exciting for translational research because they can be extracted easily, are resistant to molecular degradation and can be quantified [8].

Recent studies have identified miRNAs as novel prognostic biomarkers in various cancer types. For example, decreased miRNA-193b expression is associated with poor overall survival (OS) of colorectal cancer patients [9]. Serum miRNA-147 levels are to diagnose human non-small cell lung cancer [10]. MiRNA-200a/c was the most dysregulated miRNAs in epithelial ovarian cancer (EOC) with diagnostic and prognostic biomarker potential [11].

Since cervical cancer patients that belong to the same clinical stage have markedly different outcomes, there is an urgent need to develop more accurate and efficient prognostic biomarkers [12, 13]. Therefore, we performed a meta-analysis to investigate the prognostic value of miRNAs in cervical cancer.

## RESULTS

### Study characteristics

**Figure 1:**
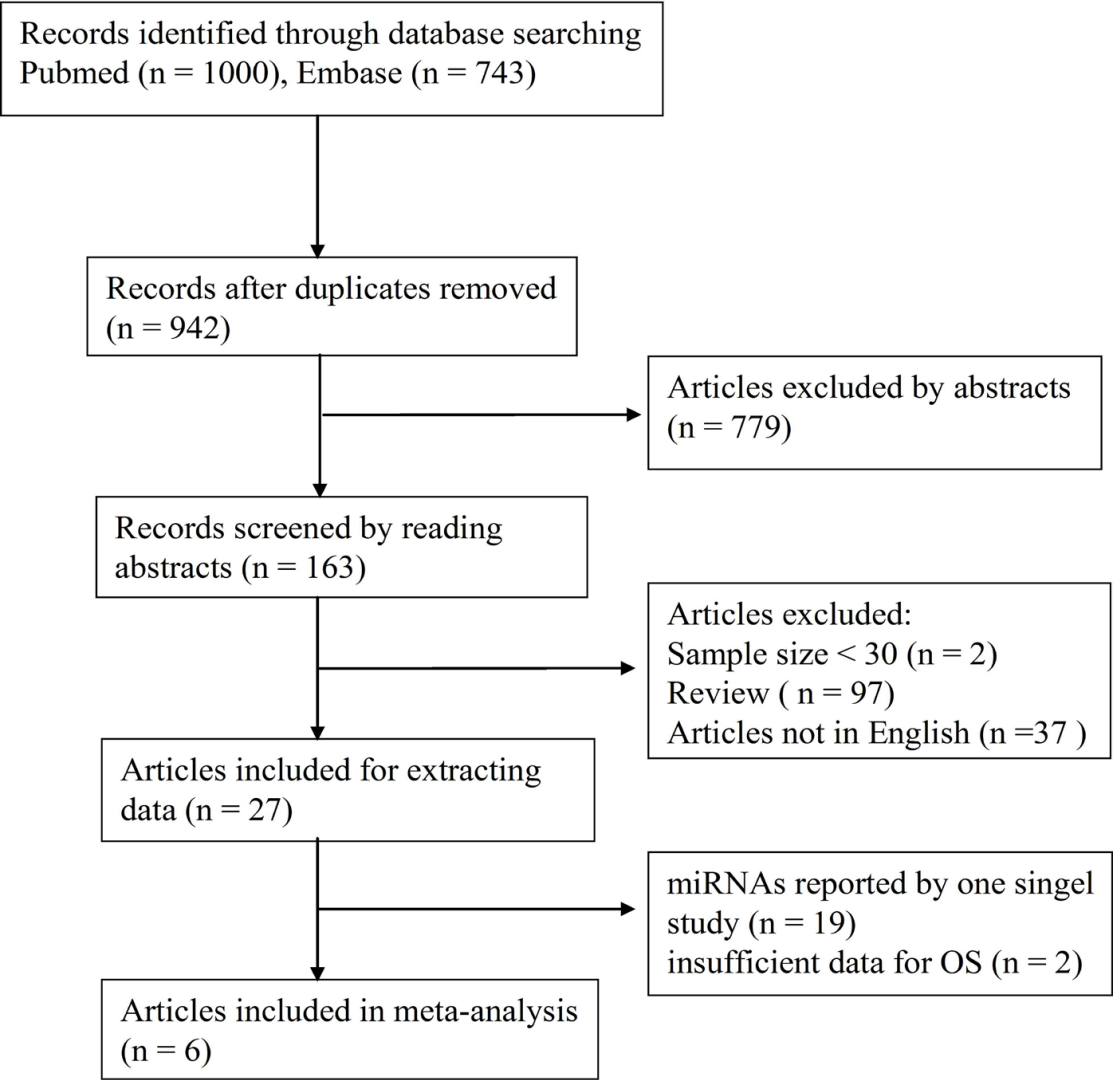
Flow chart of the meta-analysis After screening, six articles were left for further meta-analysis.

A flow diagram of the study selection process is summarized in Figure 1. In this meta-analysis, we enrolled six articles published between 2012 and 2016, which were retrospective case-control studies regarding three miRNAs [17–22] and involved 445 participants (Figure 1). In all the studies, quantitative real-time polymerase chain reaction (qRT-PCR) was employed to detect miRNAs, although the cutoff values varied. The patients in the included studies belonged to clinical stages I–IV based on the International Federation of Gynecology and Obstetrics (FIGO) classification. None of the patients received any kind of therapy such as radiotherapy and/or chemotherapy before sample collection. Each of the three miRNAs was analyzed by at least two studies. All essential characteristics of included studies (Table 1–2) were carefully investigated for the meta-analysis. There was good agreement between the reviewers according to the quality assessment of all the enrolled studies as shown in Supplementay Table 1.

**Table 1:** The main characteristics of included studies

Author (Year)	Country	miRNA	miRNA expression		FIGO stage		Histology		Lymph node metastasis		Sample type
			low	high	early	advanced	Squamous	Non-squamous	No	Yes	
**S.Azizmohammadi** **(2016)**	Iran	miRNA-145	18	17	20	15	unknown	unknown	21	14	snap-frozen tissue
**Fan (2015)**	China	miRNA-125a	30	25	26	29	50	5	26	29	snap-frozen tissue
**Wang (2015)**	China	miRNA-145	63	51	58	56	114	0	43	71	snap-frozen tissue
**Liu (2015)**	China	miRNA-196a	67	38	74	31	84	21	68	37	serum
**Hou (2013)**	China	miRNA-196a	46	46	88	4	unknown	unknown	81	11	snap-frozen tissue
**Huang (2012)**	China	miRNA-125b	40	4	36	8	0	44 (SCCC)	30	14	FFPE
**Huang (2012)**	China	miRNA-145	18	26	36	8	0	44 (SCCC)	30	14	FFPE

FFPE: formalin-fixed paraffin embedded; SCCC: neuroendocrine small cell cervical carcinoma; FIGO: International Federation of Gynecology and Obstetrics; early: FIGO stage IA-IIA, advanced : FIGO stage IIB–IV.

**Table 2:** Prognostic features and their potential targets of miRNAs

Author (Year)	Cut-off value	Follow- up (month)	miRNA	Sample size	Reference	OS		Potential targets	Expression associates with poor prognosis
						HR (95% CI)	*P*		
S. Azizmohammadi (2016)	Median (NA)	60	miRNA-145	35	L:H	a 2.62 (1.134–6.362)	0.031	_	Low
Fan (2015)	Mean (NA)	42	miRNA-125a	55	H:L	b 0.691 (0.418–1.141)	_	STAT3	Low
Wang (2015)	Median (2.5)	median 47	miRNA-145	114	H:L	a 0.63 (0.54–0.83)	0.008	_	Low
Liu (2015)	Mean (3.880)	80	miRNA-196a	105	H:L	a 3.510 (1.961–6.874)	0.025	_	high
Hou (2013)	Median (NA)	median 45.6 (1.2–60)	miRNA-196a	92	L:H	b 0.266 (0.028–2.542)	_	FOXO1 and p27Kip1	high
Huang (2012)	Mean (3.871)	mean 23.6	miRNA-125b	44	H:L	a 0.352 (0.102–1.014)	0.057	_	low
Huang (2012)	Mean (8.941)	mean 23.6	miRNA-145	44	H:L	c 0.58 (0.32–1.05)	0.072	_	low

NA, not available; L, low expression of miRNA; H, high expression of miRNA; ^a^ extracted from multivariate analyses; ^b^ estimated base on Kaplan-Meier survival curves; ^c^ extracted from univariate analyses.

### Association between the miRNAs and cervical cancer prognosis

**Figure 2:**
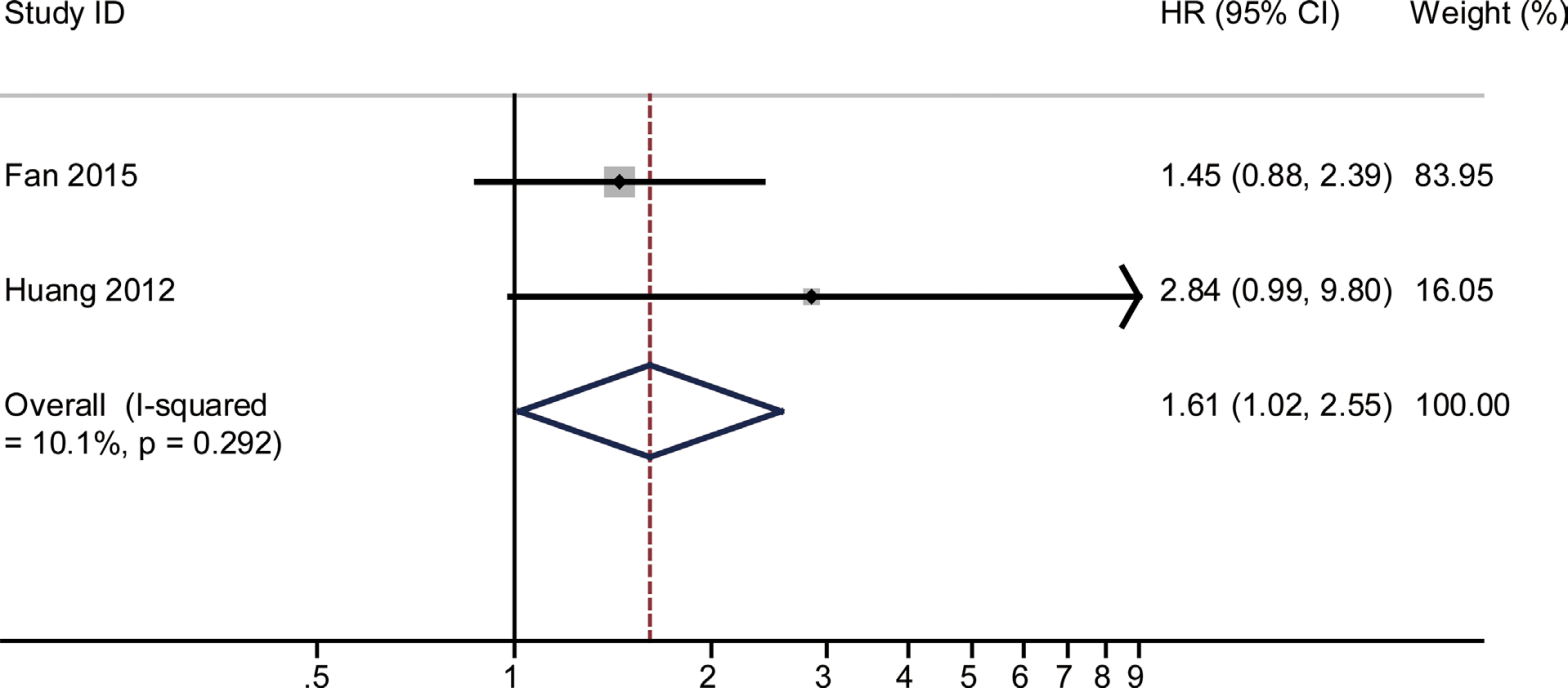
The pooled HR (hazard ratio) with overall survival among cervical cancer patients, when low expression of miRNA-125 was compared with high expression The summary estimates were obtained by using a fixed-effects model. The data markers indicate the HRs comparing low expression of miRNA-125 with high expression The size of the data markers indicates the weight of the study, which is the inverse variance of the effect estimate. The diamond data marker indicates the pooled HRs CI indicates confidence interval.

**Figure 3:**
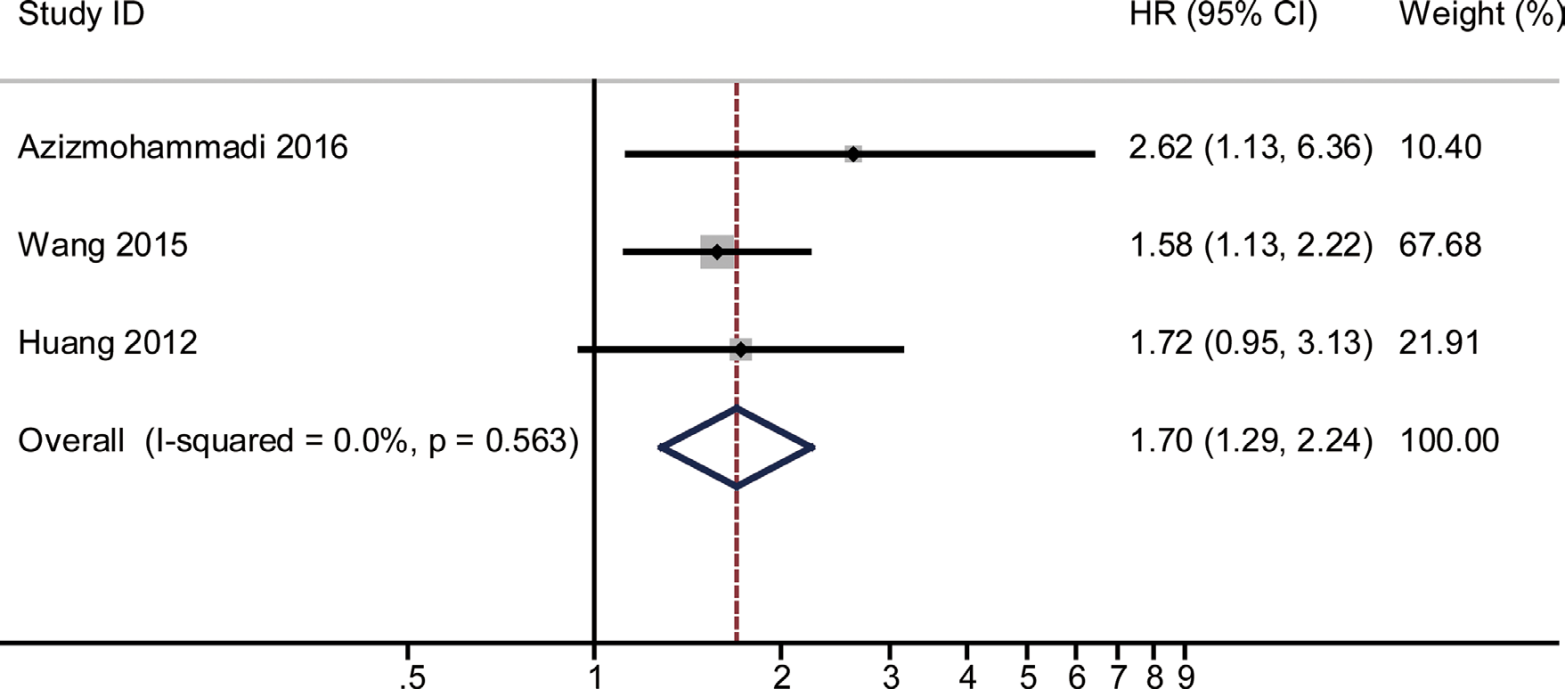
Funnel plots for detection of publication bias The pseudo 95% confidence interval (CI) is computed as part of the analysis that produces the funnel plot, and corresponding to the expected 95% CI for a given standard error (SE). HR indicates hazard ratio.

**Figure 4:**
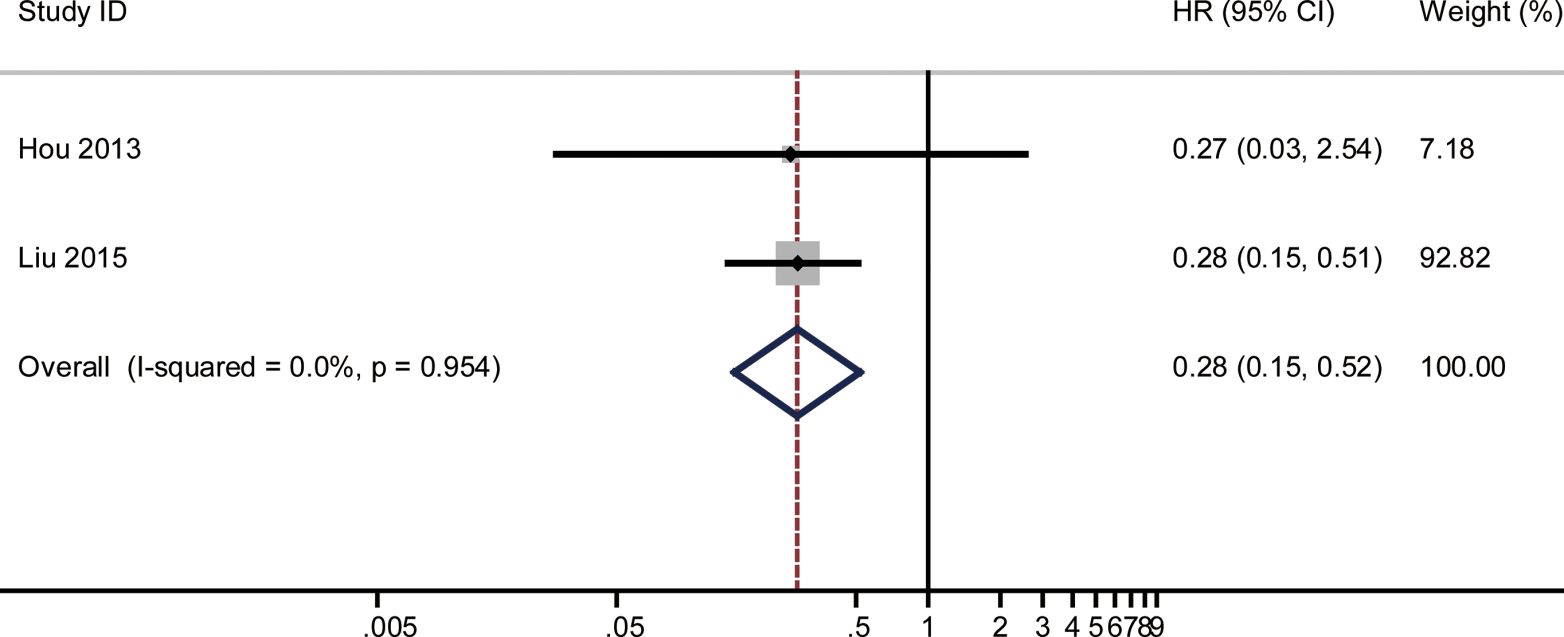
The pooled HR (hazard ratio) with overall survival among cervical cancer patients, when low expression of miRNA-145 was compared with high expression The summary estimates were obtained by using a fixed-effects model. The data markers indicate the HRs comparing low expression of miRNA-145 with high expression. The size of the data markers indicates the weight of the study, which is the inverse variance of the effect estimate. The diamond data markers indicate the pooled HRs. CI indicates confidence interval.

The combined HR and their corresponding 95% CI were calculated for miR-125, -145 and -196 and were analyzed by forest plot. Forest plot analysis showed no obvious heterogeneity for miR-125 (I^2^ = 10.1%, *P* = 0.292), miR-145 (I^2^ = 0.0%, *P* = 0.563) and miR-196 (I^2^ = 0% and *P* = 0.954), respectively. Therefore, the fixed-effects model was used for further analysis. Our analysis demonstrated that poor overall survival (OS) of cervical cancer was associated with low miR-125 (HR = 1.61, 95% CI: 1.02–2.55, *P* = 0.042; *n* = 99; Figure 2), low miR-145 (HR = 1.7, 95% CI: 1.29–2.24, *P* < 0.001; *n* = 193; Figure 3) and high miR-196 (HR = 0.28, 95% CI: 0.15–0.52, *P* < 0.001; *n* = 197; Figure 4) expression.

### Publication bias

**Figure 5:**
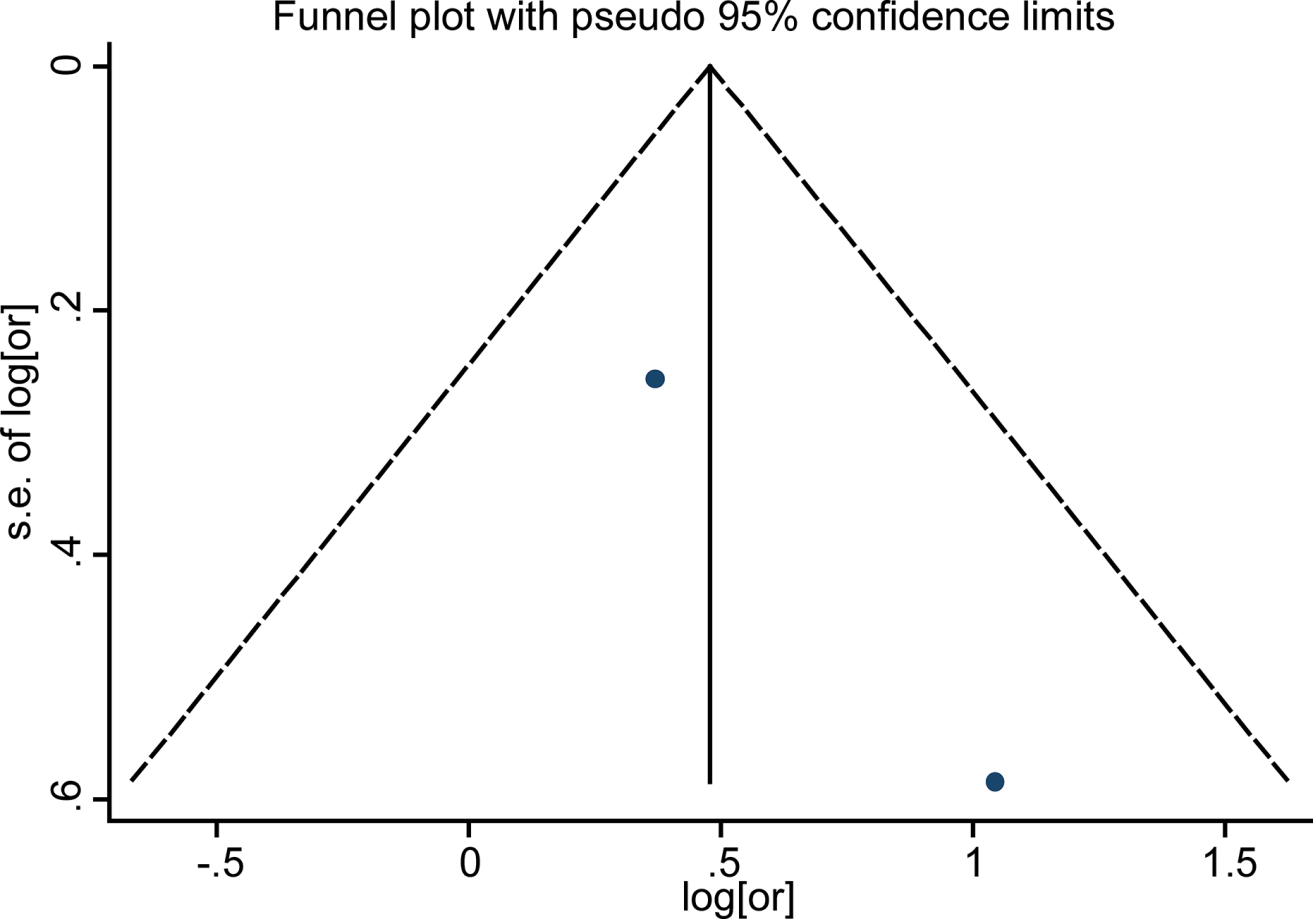
Funnel plots for detection of publication bias The pseudo 95% confidence interval (CI) is computed as part of the analysis that produces the funnel plot, and corresponding to the expected 95% CI for a given standard error. (SE). HR indicates hazard ratio.

**Figure 6:**
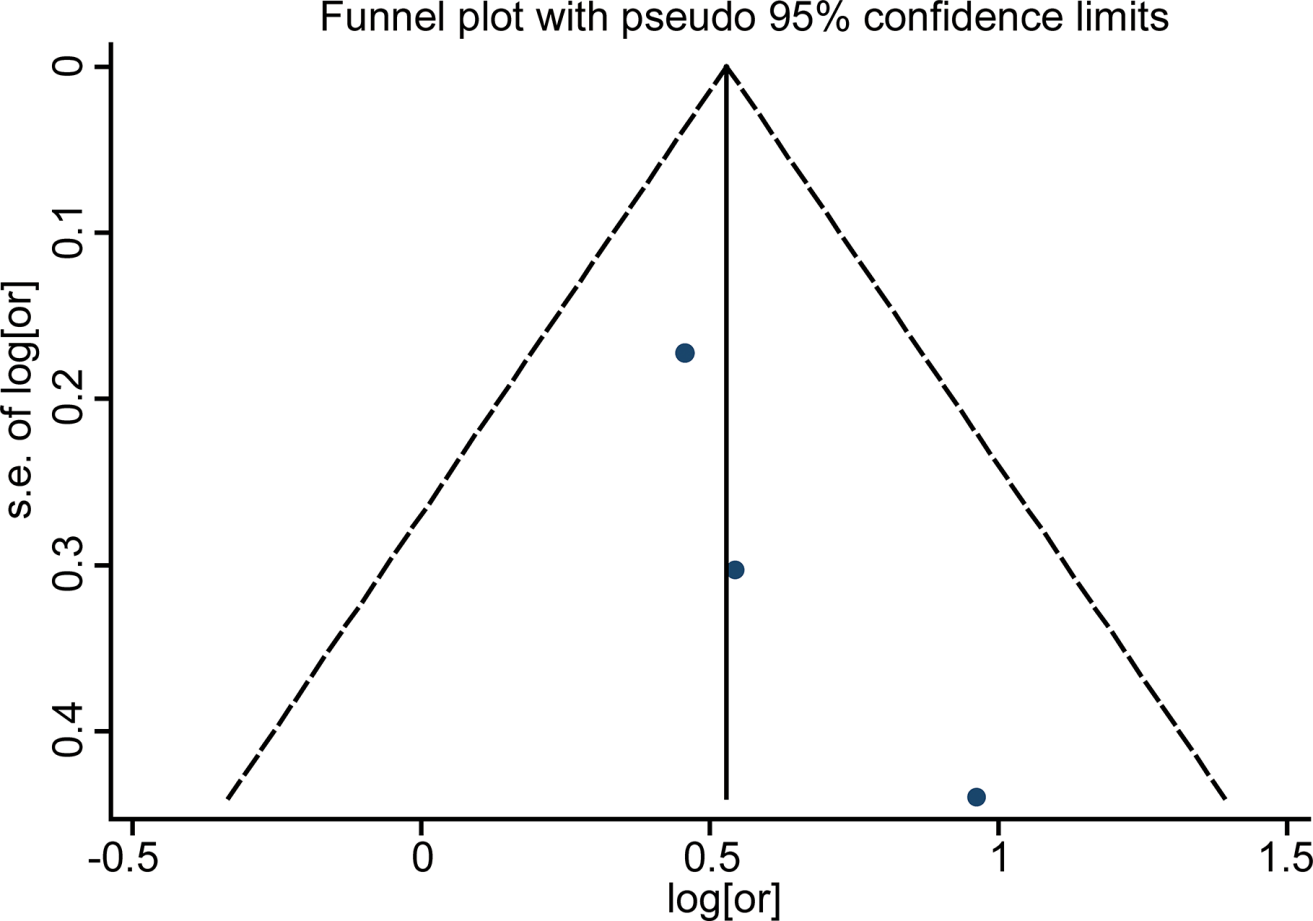
The pooled HR (hazard ratio) with overall survival among cervical cancer patients, when low expression of miRNA-196 was compared with high expression The summary estimates were obtained by using a fixed-effects model. The data markers indicate the HRs comparing low expression of miRNA-196 with high expression The size of the data markers indicates the weight of the study, which is the inverse variance of the effect estimate. The diamond data markers indicate the pooled HRs. CI indicates confidence interval.

**Figure 7:**
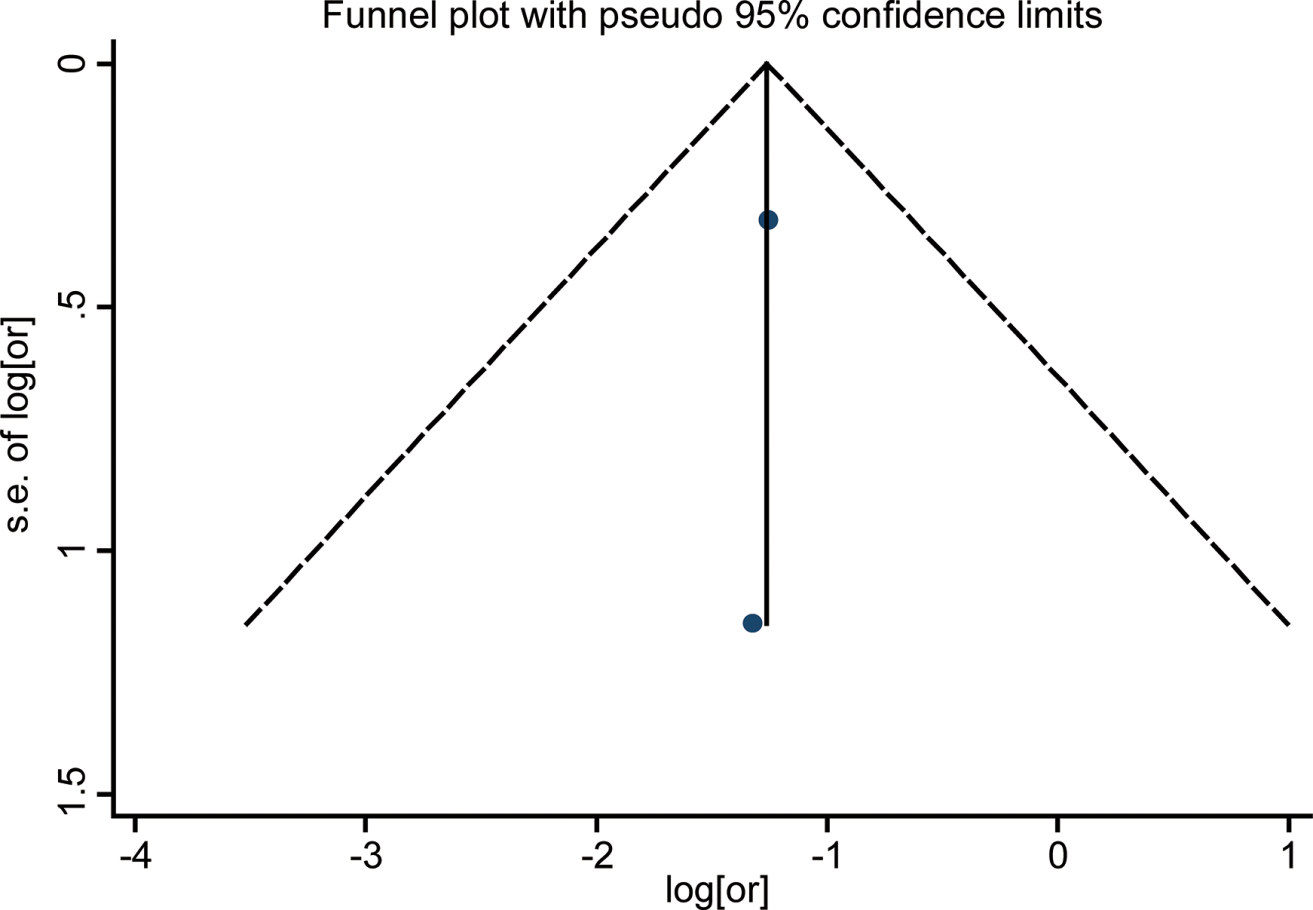
Funnel plots for detection of publication bias The pseudo 95% confidence interval (CI) is computed as part of the analysis that produces the funnel plot, and corresponding to the expected 95% CI for a given standard error (SE). HR indicates hazard ratio.

Funnel plot analysis showed no publication bias in the included studies by visual inspection (Figures 5–7). As the number of enrolled studies was limited, we abandoned the publication bias evaluation by Egger's and Begg's test.

## DISCUSSION

Our current meta-analysis showed that aberrant expression of three miRNAs, miR-125, -145 and -196 were independently associated with adverse OS of cervical cancer patients. This suggests great potential of using miRNAs in future clinical applications to treat high risk cervical cancer patients.

Previous studies have shown that FIGO stage, patient age, tumor size, parametrial infiltration, lymph-vascular space invasion, lymph node metastasis, and hemoglobin levels are good prognostic factors that determine survival of cervical cancer patients [23]. However, these clinicopathological factors alone are not sufficient to predict prognosis of cervical cancer patients. Therefore, novel molecular biomarkers are necessary to accurately predict the prognosis of this disease.

Aberrant expression of miRNAs is involved in several forms of solid tumors such as breast cancer, colorectal cancer, ovarian carcinoma, lung cancer, hepatocellular carcinoma, and genitor-urinary cancer [4, 5, 7, 9–11, 24, 25]. They act as tumor promoters or suppressors based on the function of their target genes. More importantly, a single miRNA can regulate the expression of multiple genes because of their ability to bind to their mRNA targets [26]. MiRNAs directly regulate at least 30% of the genes in a cell [27]. Therefore, miRNA expression profiles can be used to determine tumor progression, prognosis and response to specific cancer therapies.

Furthermore, two studies included in this meta-analysis characterized the functional role of miRNAs in cervical cancer cells. Hou and colleagues showed that miR-196a directly targeted FOXO1 and p27Kip1, two key effectors of PI3K/Akt signaling; when overexpressed, miR-196a increased proliferation and G1/S-phase transition of cervical cancer cells whereas its suppression had the opposite effect [21]. Fan and colleagues demonstrated that high miR-125a expression suppressed the growth, invasion and epithelial-mesenchymal transition (EMT) of cervical cancer cells both *in vivo* and *in vitro* by reducing STAT3 expression; it also conferred G2/M cell cycle arrest by inhibiting several G2/M checkpoint proteins [18]. These data highlight the importance of miR-196a and miR-125a in the growth and progression of cervical cancer.

An earlier meta-analysis of 19 studies on the same topic showed that decreased miRNA expression was an indicator of poor prognosis in cervical cancer patients [28]. Compared to the meta-analysis by Dai S and colleagues, our meta-analysis had some advantages. First, no statistically significant heterogeneity was found among the results of individual studies (Figures 2, 3, 4). In the earlier meta-analysis, substantial heterogeneity was thought to be present among the studies assessing the prognostic performance of miRNAs (OS: I^2^ = 85.6%, *P* < 0.001). Second, due to the lack of related research focusing on the same miRNA, the earlier meta-analysis calculated the pooled effect of different miRNAs for clinical evaluation and the conclusion drawn remained preliminary. Meanwhile, we chosen those miRNAs which published at least 2 times and performed subgroup analysis based on specific miRNA. Finally, our results are all of notable significance, suggesting the sample enrolled in this meta-analysis was relatively sufficient.

Despite these advantages, some limitations of our meta-analysis should be acknowledged which are as follows: (1) the seven included studies varied in the cancer pathological type that were analyzed. In three studies, the main cancer type was squamous cell carcinoma; two studies had no clear histological classification of cancer type; one publication studied patients with rare neuroendocrine small cell cervical carcinoma (SCCC); (2) there were only seven eligible studies in this meta-analysis in regard to OS; (3) the samples were either FFPE (formalin-fixed paraffin embedded), fresh-frozen or serum specimens. While six studies tested the miRNA expression in tumor tissues, one detected miRNAs in the serum; (4) studies enrolled in this meta-analysis were mostly conducted in Asia. Therefore, additional studies are required in other populations. While we identified 3 miRNAs that are associated with the prognosis of cervical cancer in this meta-analysis, it was hard to assess the significance of their correlation with cervical cancer because they have been reported only few times. Large prospective studies are needed to validate the prognostic values of miRNAs in homogeneous cervical cancer patients.

In conclusion, our meta-analysis shows 3 miRNAs, miR-125, -145 and -196 with prognostic potential to predict OS in cervical cancer patients.

## MATERIALS AND METHODS

### Search strategy

We searched the PUBMED and EMBASE online databases to identify relevant studies with the keywords microRNA or miRNA or MIR and cervical cancer or cervical carcinoma or uterine cervix cancer. The inclusion criteria were: (1) the study was conducted in human subjects; (2) the studies were case-controlled and examined the prognostic significance of miRNA in cervical cancer patients in regard to overall survival (OS); (3) the data on the miRNAs included that of patients and controls; and (4) article was published in English as a full manuscript and was not a meeting abstract or review. We focused on miRNAs that were analyzed in at least 2 studies. Based on these criteria, 6 papers involving three miRNAs were selected for the meta-analysis (Figure 1).

### Data extraction

We collected the first author's last name, publication year, country of origin, sample size, cut-off value, follow-up duration, miRNA detection method, endpoints and survival data from all studies. Two reviewers (Z Chen and Y Han) independently extracted the data. Any discrepancies were resolved by discussion with senior reviewers.

### Assessment of methodologic quality

Two reviewers independently evaluated the quality of all the included studies with the Newcastle–Ottawa Scale (NOS) (Wells et al., 2009) for case-control studies. The NOS consists of three sections: selection, comparability, and exposure. The NOS assigns a maximum score of 4 for selection, 2 for comparability, and 3 for exposure. Hence, a score of 9 indicates the highest quality.

### Statistical analysis

To statistically assess the prognostic effects of miRNAs on the survival of cervical cancer, we extracted individual HRs and associated 95% CIs that were available. Otherwise, they were estimated from the survival data or Kaplan-Meier survival curves using methods suggested by Parmar et al. [14] and Tierney et al. [15]. In addition, when HRs were available from both univariate and multivariate analyses, the latter were preferred because multivariate analyses considered possible confounding effects [16]. In general, a HR > 1 indicated a poor outcome for the patient with reduced expression of miRNAs. Forest plots were employed to illustrate the HR and its 95% CI for each of the included studies as well as the combined result. The Cochrane's Q statistic and I^2^ statistic were computed to test the significance of potential heterogeneity. If studies reported moderate or low heterogeneity (I^2^ < 50%), the fixed-effects model was used for pooling. Otherwise, the random-effects model was adopted for I^2^ 50%. Publication bias was evaluated by visual inspection of funnel plots. *P* < 0.05 was considered statistically significant. All statistical analyses were performed with STATA version 11 software (Stata Corp, College Station, TX).

## SUPPLEMENTARY MATERIALS TABLE



## References

[1] Torre LA, Bray F, Siegel RL, Ferlay J, Lortet-Tieulent J, Jemal A (2015). Global cancer statistics, 2012. CA Cancer J Clin.

[2] Arbyn M, Castellsague X, de Sanjose S, Bruni L, Saraiya M, Bray F, Ferlay J (2011). Worldwide burden of cervical cancer in 2008. Ann Oncol.

[3] Li S, Hu T, Lv W, Zhou H, Li X, Yang R, Jia Y, Huang K, Chen Z, Wang S, Tang F, Zhang Q, Shen J (2013). Changes in prevalence and clinical characteristics of cervical cancer in the People's Republic of China: a study of 10,012 cases from a nationwide working group. Oncologist.

[4] Chang JT, Wang F, Chapin W, Huang RS (2016). Identification of MicroRNAs as Breast Cancer Prognosis Markers through the Cancer Genome Atlas. PLoS One.

[5] Medina Villaamil V, Santamarina Cainzos I, Aparicio Gallego G, Quindos Varela M, Dopico Vazquez D, Antolin Novoa S, Garcia Campelo R, Reboredo M, Valladares Ayerbes M, Anton Aparicio LM (2011). MicroRNAs as emerging biomarkers for micrometastasis detection in genitourinary tumors. J Clin Oncol.

[6] Liu B, Ding JF, Luo J, Lu L, Yang F, Tan XD (2016). Seven protective miRNA signatures for prognosis of cervical cancer. Oncotarget.

[7] O'Bryan S, Dong S, Mathis JM, Alahari SK (2016). The roles of oncogenic miRNAs and their therapeutic importance in breast cancer. Eur J Cancer.

[8] Turchinovich A, Weiz L, Langheinz A, Burwinkel B (2011). Characterization of extracellular circulating microRNA. Nucleic Acids Res.

[9] Guo F, Luo Y, Mu YF, Qin SL, Qi Y, Qiu YE, Zhong M (2016). miR-193b directly targets STMN1 and inhibits the malignant phenotype in colorectal cancer. Am J Cancer Res.

[10] Chu G, Zhang J, Chen X (2016). Serum level of microRNA-147 as diagnostic biomarker in human non-small cell lung cancer. J Drug Target.

[11] Teng Y, Su X, Zhang X, Zhang Y, Li C, Niu W, Liu C, Qu K (2016). miRNA-200a/c as potential biomarker in epithelial ovarian cancer (EOC): evidence based on miRNA meta-signature and clinical investigations. Oncotarget.

[12] Rossi PJ, Horowitz IR, Johnstone PA, Jani AB (2009). Lymphadenectomy for patients with cervical cancer: is it of value?. J Surg Oncol.

[13] Kim HS, Kim JH, Chung HH, Kim HJ, Kim YB, Kim JW, Park NH, Song YS, Kang SB (2011). Significance of numbers of metastatic and removed lymph nodes in FIGO stage IB1 to IIA cervical cancer: Primary surgical treatment versus neoadjuvant chemotherapy before surgery. Gynecol Oncol.

[14] Parmar MK, Torri V, Stewart L (1998). Extracting summary statistics to perform meta-analyses of the published literature for survival endpoints. Stat Med.

[15] Tierney JF, Stewart LA, Ghersi D, Burdett S, Sydes MR (2007). Practical methods for incorporating summary time-to-event data into meta-analysis. Trials.

[16] Mallett S, Royston P, Dutton S, Waters R, Altman DG (2010). Reporting methods in studies developing prognostic models in cancer: a review. BMC Med.

[17] Huang L, Lin JX, Yu YH, Zhang MY, Wang HY, Zheng M (2012). Downregulation of six microRNAs is associated with advanced stage, lymph node metastasis and poor prognosis in small cell carcinoma of the cervix. PLoS One.

[18] Fan Z, Cui H, Xu X, Lin Z, Zhang X, Kang L, Han B, Meng J, Yan Z, Yan X, Jiao S (2015). MiR-125a suppresses tumor growth, invasion and metastasis in cervical cancer by targeting STAT3. Oncotarget.

[19] Wang Q, Qin J, Chen A, Zhou J, Liu J, Cheng J, Qiu J, Zhang J (2015). Downregulation of microRNA-145 is associated with aggressive progression and poor prognosis in human cervical cancer. Tumour Biol.

[20] Azizmohammadi S, Safari A, Kaghazian M, Sadrkhanlo M, Yahaghi E, Farshgar R, Seifoleslami M (2016). Molecular identification of miR-145 and miR-9 expression level as prognostic biomarkers for early-stage cervical cancer detection. QJM.

[21] Hou T, Ou J, Zhao X, Huang X, Huang Y, Zhang Y (2014). MicroRNA-196a promotes cervical cancer proliferation through the regulation of FOXO1 and p27Kip1. Br J Cancer.

[22] Liu P, Xin F, Ma CF (2015). Clinical significance of serum miR-196a in cervical intraepithelial neoplasia and cervical cancer. Genet Mol Res.

[23] Biewenga P, van der Velden J, Mol BW, Stalpers LJ, Schilthuis MS, van der Steeg JW, Burger MP, Buist MR (2011). Prognostic model for survival in patients with early stage cervical cancer. Cancer.

[24] Shen S, Lin Y, Yuan X, Shen L, Chen J, Chen L, Qin L, Shen B (2016). Biomarker MicroRNAs for Diagnosis, Prognosis and Treatment of Hepatocellular Carcinoma: A Functional Survey and Comparison. Sci Rep.

[25] Alhasan AH, Scott AW, Wu JJ, Feng G, Meeks JJ, Thaxton CS, Mirkin CA (2016). Circulating microRNA signature for the diagnosis of very high-risk prostate cancer. Proc Natl Acad Sci U S A.

[26] Chen K, Rajewsky N (2007). The evolution of gene regulation by transcription factors and microRNAs. Nat Rev Genet.

[27] Lewis BP, Burge CB, Bartel DP (2005). Conserved seed pairing, often flanked by adenosines, indicates that thousands of human genes are microRNA targets. Cell.

[28] Dai S, Lu Y, Long Y, Lai Y, Du P, Ding N, Yao D (2016). Prognostic value of microRNAs in cervical carcinoma: a systematic review and meta-analysis. Oncotarget.

