# Supplementary material for: Systematic review and meta-analysis of the prognostic significance of microRNAs in cervical cancer
Source: Oncotarget. 2017 Dec 16;9(24):17141–8. doi: 10.18632/oncotarget.23839 (PMC5908312; doi:10.18632/oncotarget.23839)
Supplement: Supplementary file 1 [file oncotarget-09-17141-s001.pdf]

Systematic review and meta-analysis of the prognostic significance of microRNAs in cervical cancer

SUPPLEMENTARY MATERIALS

Supplementary Table 1: Results of analysis with NOS for included studies

Author (Year)	Selection				Comparability		Outcome			Total score
	a	b	c	d	e	f	g	h	i	
S. Azizmohammadi (2016)	1	1	1	1	1	0	1	1	0	7
Fan (2015)	1	1	1	1	1	1	1	1	0	8
Wang (2015)	1	1	1	1	1	1	1	1	1	9
Liu (2015)	1	1	1	1	1	1	1	1	0	8
Hou (2013)	1	1	1	1	1	0	1	1	1	8
Huang (2012)	1	1	1	1	1	1	1	1	1	9
Huang (2012)	1	1	1	1	1	1	1	1	1	9

a = Adequate definition of the case (1 star); b = Representativeness of the cases (1 star); c = Selection of Controls; d = Definition of Controls (1 star); e = Study controls for age (1 star); f = Study controls for any additional factor (1 star); g = Ascertainment of exposure; h = Same method of ascertainment for cases and controls; i = Non-Response rate(1 star).
